# The relationship between microbial community vitality and ATP bioburden in bottom waters under fuel microcosms

**DOI:** 10.1099/acmi.0.000411

**Published:** 2023-04-26

**Authors:** Frederick Jay Passman, Jordan Schmidt, Russell P. Lewis

**Affiliations:** ^1^​ Biodeterioration Control Associates, Inc., PO Box 3659, Princeton, NJ 08643-3659, USA; ^2^​ LuminUltra Technologies, Ltd, 520 King Street, Fredericton, NB, E3B 6G3, Canada; ^3^​ Marathon Petroleum Company, LP, 11631 US Route 23, Catlettsburg, KY 41129, USA

**Keywords:** adenosine triphosphate, ATP, adenosine diphosphate, ADP, adenosine monophosphate, AMP, adenylate energy charge, AEC, diesel fuel, microbiology

## Abstract

Adenylate energy charge (AEC) – computed from the ATP, ADP and AMP concentrations in a specimen – reflect the net physiological state of the microbial population in that specimen. Previous research has demonstrated that healthy microbial populations maintain AEC≥0.8. As populations are subjected to stresses, or – in closed systems – deplete the available nutrients, respond to the accumulation of toxic metabolites, or both, AEC decreases (often to <0.5). Aqueous-phase samples from a set of fuel–water microcosms were tested for cellular ATP (cATP) and AEC. This paper reports on the precision of the AEC test method and the relationship between cellular AEC and cATP bioburdens in the aqueous phase of fuel over aqueous-phase microcosms.

## Data Summary

The authors confirm all supporting data and protocols have been provided within the article or through supplementary data files. Four supplementary tables are available with the online version of this article. Tables S1–S4 contain the raw data, in log_10_(pg m^l−1^), or AEC, from which Table 4 summary statistics were computed.

## Introduction

Atkinson and Walton [1] proposed the use of adenylate energy charge (AEC) as a parameter for estimating the physiological state of cell populations. According to Atkinson and Walton:

(1) $$AEC=\frac{\left[ATP\right]+0.5\left[ADP\right]}{\left[ATP\right]+\left[ADP\right]+\left[AMP\right]}$$

where [ATP], [ADP] and [AMP] are the concentrations of ATP, ADP and AMP, respectively. As reviewed by Karl [2], between 1967 and 1980 hundreds of studies validated the general utility of AEC. The consensus was that AEC:

- provided a linear indication of the population’s metabolic energy,
- generally appeared to regulate overall metabolism, and
- was invariably ≥0.8 when biosynthesis, proliferation or both were observed.

Notwithstanding the apparent enthusiasm for AEC, some authors [3–5] cited its limitations. Lowery *et al*. [3] argued that AEC was an insensitive metabolic indicator. Others argued that while AEC could be useful for monitoring the metabolic state of pure cultures growing in broth media, it was insufficiently granular for natural populations. First, the total nucleotide concentration per cell varied among taxa. Second, within any natural population some cells were likely to be metabolically active, others dormant and yet others moribund. Moreover, in the 1970s and 1980s, neither nucleotide extraction nor detection had been standardized.

Despite these limitations, Holm-Hansen and Karl reported that AEC was a useful parameter for assessing the overall health of marine microbiomes [6]. By the mid-1980s interest in AEC seems to have waned. Searches of online citation indices did not identify any papers published between 1985 and 2014. De Fuente *et al.* [7] appear to have sparked renewed interest in AEC as a tool for assessing the physiological state of microbiomes.

Reviewing two decades of fuel microbiology literature, Passman [8] noted that it was common to recover substantial microbial bioburdens from fuel systems that had little evidence of biodeterioration and that, conversely, substantial biodeterioration could be observed in systems with apparently minimal bioburdens. The relative biodeteriogenicity of contaminant populations was only one of several reasons for these observations. The heterogeneous distribution of microbes in fuel systems could also account for the weak correlation between bioburdens and severity of biodeterioration damage [9]. It is also possible that the culturability of microbes in test specimens could differ substantially from their metabolic state *in situ*. Microbes dormant in fuels were likely to become metabolically active once transferred to a nutrient-rich growth medium. Conversely, a substantial percentage of microbes that are metabolically active *in situ* will not grow in or on the most commonly used nutrient media. Recently, Passman *et al.* [10] evaluated the impact of microbicides and non-microbicidal additives in water-miscible metalworking fluids. They found that AEC reliably differentiated between effective and ineffective dosing, and demonstrated the adverse impact that putatively non-biocidal, biostable additives had on microbial populations. Thus, AEC might be a useful tool for assessing the metabolic state of microbes in fuel system specimens.

In 2016, members of the Coordinating Research Council, Inc. (CRC) Fuel Corrosivity Panel designed a multivariate laboratory study to provide a basis for assessing the primary factors contributing to retail diesel fuel system corrosion. A 128-microcosm laboratory study was commissioned as CRC Project DP-07-16-1 [11]. At the end of the microcosm study, the authors had an opportunity to test a limited number of samples for cellular ATP, ADP and AMP ([cATP], [cADP] and [cAMP] – collectively [AXP]). The purpose of this additional testing was to assess the potential utility of AEC for assessing fuel system microbiome biodeteriogenicity. This paper presents the AXP data and AEC ratios.

## Methods

### Test plan

Members of the CRC Fuel Corrosivity Panel designed a 128-microcosm test plan that included the following variables:

**Table:** 

Fuel grade – LSD; ULSD, 500 ml	Monoacid lubricity additive (MAL) – none or 200 mg l^–1^
FAME – none or 5 % by volume	Cold flow improver (CFI) – none or 200 mg l^–1^
Ethanol – none or 10 000 mg l^–1^	Corrosion inhibitor (CI) – none or 8–10 mg l^–1^
Glycerin – none or 5000 mg l^–1^	Conductivity additive (CA) – none or 2–3 mg l^–1^
Free water – none or 250 ml	Microbial inoculum – added or not

The subset included in the AXP protocol evaluation is listed in Table 1. The microcosms contained a 250 ml aqueous phase under either low sulphur diesel (LSD) or ultra-low-sulphur diesel (ULSD).

**Table 1.** Microcosm properties; AXP test specimen sources*

Microcosm	Composition									
	S-grade	B5†	Inoculum‡	Glycerol	Ethanol	MAL	CFI	CI	CA	PR§
A	LSD	−¶	+	+	−	−	+	−	−	+
B	LSD	+	+	+	−	−	+	−	−	+
C	ULSD	−	+	−	−	−	−	−	−	+
D	ULSD	−	+	+	−	+	−	−	−	+
E	ULSD	−	−	+	+	−	−	−	−	+
F	ULSD	−	−	−	+	+	+	−	−	+
G	ULSD	+	−	+	−	+	−	−	+	−
H	LSD	+	+	+	+	+	+	−	+	−

*All eight microcosms had an aqueous-phase and all specimens for AXP testing were from aqueous-phase samples.

†B5 – FAME added to produce B% biodiesel.

‡Challenge population added (+) or not (−).

§PR – polymeric epoxy resin coupon.

¶“−”: component not present; “+”: component present.

**Fig. 1.**
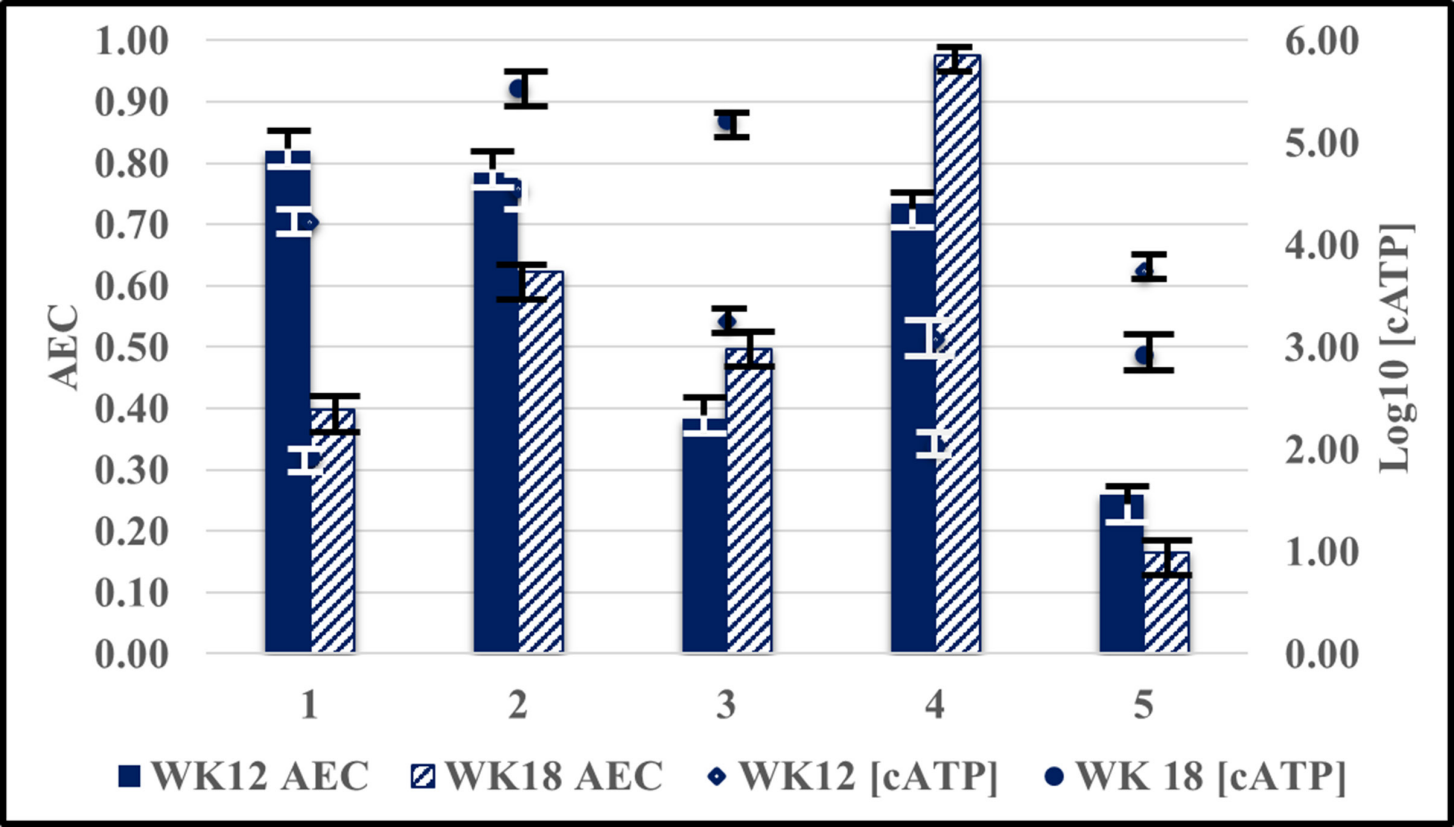
Fuel over Bushnell-Hass medium microcosms – log_10_ [cATP] and AEC changes between weeks 12 and 18.

All microcosms included four carbon steel corrosion coupons (Fig. 1). Polymeric resin coupons were suspended in a subset of microcosms. All microcosms were stored in the dark, in fume hoods, at laboratory room temperature (20±2 °C).

### Inoculum

An inoculum was prepared from Underground storage tank (UST) samples. Initially primary microcosms of either LSD or ULSD over Bushnell-Hass medium [12] were inoculated with bottom-water samples from microbially contaminated ULSD UST. Once the aqueous-phase ATP bioburdens were >4log_10_ pg ml^−1^, they were pooled and used to inoculate secondary fuel over Bushnell-Hass medium microcosms. At the beginning of microcosm studies (T_0_), the challenged test microcosms were inoculated with 1 ml of secondary bottom-water in which the ATP bioburden was 4.5±0.5log_10_ pg ml^−1^.

### AXP testing

Aqueous-phase specimens were collected and extracted as per ASTM D7687 [13]. The method was modified in that 1 ml of the aqueous specimens was tested instead of 5 ml water specimens prescribed in D7687. Briefly, the specimen was filtered through a 0.7 µm glass fibre filter to capture cells. Interfering material was then washed away using a proprietary rinsing agent (LumiClean; LuminUltra Technologies) and air-dried using a 60 ml syringe. The washed and dried cells were then lysed using 1.0 ml of a proprietary lysing agent (UltraLyse 7; LuminUltra Technologies) and flushed into 9.0 ml of a proprietary buffer (UltraLute; LuminUltra Technologies). Proprietary ATP enzyme, ADP enzyme and AMP enzyme reagents were calibrated using 1 ng ml^−1^ ATP, ADP and AMP reference standard solutions, respectively. Additionally, the ADP enzyme and AMP enzyme reagents were tested with the ATP reference standard solution. For each calibration test, 100 µl of enzyme reagent was mixed with 100 µl of reference standard in a 12×55 mm reaction tube. The tube was placed into a PhotonMaster luminometer and luminescence was recorded as relative light units (RLU). As illustrated in Table 2, calibration data were entered into an Excel worksheet. For each specimen, 100 µl of extract was transferred to each of three 12×55 mm reaction tubes. Next, 100 µl of ATP enzyme reagent was added to the first tube and RLU were measured. The RLU determinations for ATP+ADP and ATP+AMP were determined analogously by reacting 100 µl of the ADP enzyme reagent with the extract in the second tube and 100 µl of the AMP enzyme reagent with the extract in the third tube.

**Table 2.** AXP reagent calibration data

Calibration with ATP standard			Calibration with ADP or AMP standard	
ATP enzyme +ATP standard (RLU_ATP1_)	ADP enzyme +ATP standard (RLU_ATP2_)	AMP enzyme +ATP standard (RLU_ATP3_)	ADP enzyme +ADP standard (RLU_ADP1_)	AMP enzyme +AMP standard (RLU_AMP1_)
1906	1973	2153	1265	1165

For each sample, 100 µl was reacted with each of the AXP enzyme reagents. AEC is computed from equation 1 above and:

(2) $$\left[cATP]=(RLU_{ATP}÷RLU_{ATP1})(10,000÷sample\;vol\;(mL)\right)$$

(3) $$\left([cAMP]+[cATP])=(RLU_{AMP+ATP}÷RLU_{ATP3})(10,000÷sample\;vol\;(mL)\right)$$

(4) $$\left([cAMP]+[cATP])=(RLU_{AMP+ATP}÷RLU_{ATP3})(10,000÷sample\;vol\;(mL)\right)$$

(5) [cADP]=([cADP])+[cATP])−[cATP]

(6) [cAMP]=([cAMP])+[cATP])−[cATP]

### Statistical analysis

All statistics were computed using the Microsoft Excel Analysis ToolPak Add-in.

## Results and discussion

### Test method precision

A single analyst performed triplicate AXP tests on eight samples to determine the repeatability standard deviation (*s*_r_) for log_10_ [cATP], log_10_ [cADP], log_10_ [cAMP] and AEC. As shown in Table 3, the average coefficients of variation (CV) ranged from 0.7 % (log_10_ [cATP]) to 3.7 % (AEC). Within the two to three log_10_ [AXP] concentration ranges of the tested samples, *s*_r_ was not affected by log_10_ [AXP].

**Table 3.** AXP test method repeatability precision for fuel over Bushnell-Hass medium microcosm, with aqueous phase samples*

Parameter	Statistic			
	*n*	*s*_r_	Slope *s*_r[X]_	Average CV (%)
cATP	24	0.17	0.0002	0.7
cADP	24	0.34	0.052	2.6
cAMP	24	0.38	0.0002	3.6
AEC	24	0.19	0.051	3.7

*Data from which statistics were computed are found in Table S1.

### Analyte aging

The reagent’s manufacturer recommended that extracts be tested within 4 h after preparation. To test the impact of extract ageing, tests were performed within 30 min after preparation and again after 1 week of storage at ambient room temperature (18±2 °C). The results, summarized in Table 4, demonstrated that each of the analytes – and consequent AEC – were stable for at least a week when extracts were stored in the dark at room temperature. The rationale for this ageing study was to verify the conclusions drawn about ASTM D7687 [13] extracts that were prepared at Marathon Petroleum Company LLC’s Refining Analytical and Development (MPC) laboratory, tested for [cATP] and subsequently shipped to Biodeterioration Control Associates, Inc. (BCA) for retesting.

**Table 4.** AXP test method analyte ageing (all [cAXP] values are log_10_ [cAXP])

Parameter	T_1_		T_2_		*F*_OBS_	*F*_CRIT_	*P*_0.95_
	Average	*s*	Aerage	*s*			
cATP	3	1.00	2.9	0.98	0.013	4.6	0.91
cADP	2.6	0.73	2.7	0.79	0.058	4.6	0.81
cAMP	2.5	0.73	2.9	0.78	1.01	4.6	0.33
AEC	0.6	0.21	0.5	0.17	0.55	4.6	0.47

Comparison of the two data sets by Student’s *t*-test (two sided) and one-way ANOVA yielded equivocal indications of whether the differences in log_10_ [cATP] results obtained at the two labs were statistically significant (Table 3). The *t*-test statistics indicated that they were (*P*_0.95_=0.01) but the *F*-test statistics suggested that they were not (*P*_0.95_=0.13). The data summarized in Table 5 were from tests run by two different analysts, using reagents from two different sets of production lots. Consequently, any differences between the two data sets reflect the combined primary effects of:

- extract ageing during storage and shipping,

**Table 5.** Summary statistics – comparison of log_10_ [cATP] data obtained at MPC* and BCA†, respectively, 1 week apart

Statistic	MPC	BCA
Average	3	2.8
*s*	1.9	0.71
*t*_OBS_	−2.93	
*t*_CRIT_	2.08	
*P*_0.95_	0.01	
*F*_OBS_	2.40	
*F*_CRIT (1,22)_	4.10	
*P*_0.95_	0.13	

*MPC – Marathon Petroleum Company.

†BCA – Biodeterioration Control Associates.

- reproducibility variation between analysts, and
- variation due to differences in reagent ages.

Given that both labs reported average [cATP]=3log_10_ pg ml^−1^ by ASTM D7687, and that the AXP protocol also yielded an average [cATP]=3log_10_ pg ml^−1^, the ANOVA statistic, indicating that the difference between the two data sets was not statistically different at the 95 % confidence level, seems to be the more accurate assessment.

### Impact of test microcosm conditions on AEC

As explained in the Introduction, AEC was believed to provide a basis for assessing a cell population’s net physiological state. Thomas and Dawson [14] suggested that AEC≥0.8 was indicative of a robust, dynamic population and that when AEC≤0.4 the population is unable to sustain biosynthesis or proliferation. Environmental stress, including nutrient limitation and the presence of toxic chemicals at sublethal concentrations, was indicated when AEC ranged from <0.4 to <0.8.

The AXP data and their summary statistics are provided in Tables S1–S4. Among the samples collected for AXP testing, AEC ranged from 0.29±0.01 (conditions: microbiologically challenged, B5 LSD with glycerol, ethanol, mono-acid lubricity additive, cold flow improver and conductivity additive) to 0.88±0.05 (conditions: B5 ULSD with glycerol, mono-acid lubricity additive and cold flow improver). The summary statistics for all four AXP parameters are provided in Table 6. The only significant effect was that of ethanol on log_10_ [cATP]. The average log_10_ [cATP] values in microcosms with and without ethanol were 2.5±0.79 and 4.0±0.60 log_10_ pg ml^−1^, respectively (Table 6a). These results corroborated those reported by Dodos *et al.* [15]. Interestingly, the negative relationship between ethanol and [cATP] was not reflected in the respective AECs (Table 6d). It is important to note that the sample set included in this initial AXP test method assessment was small (triplicate, aqueous-phase specimens from eight microcosms). Most of the AEC results indicated that microcosm populations were moderately stressed, but biologically active and able to proliferate.

**Table 6.** Impact of microcosm chemistry on AXP

Factor	Present		Absent		*F*_OBS_	*F*_CRIT_	*P*_0.95_
	Average	*s*	Average	*s*			
**6a. Log**_**10**_ **[cATP]**							
Challenge*	3	1.1	4.1	0.51	2.20	5.12	0.17
FAME	4.0	0.72	3	1.0	0.77	5.12	0.40
Ethanol	2.5	0.79	4.0	0.60	11.8	5.12	0.007
**6b. Log_10_ [cADP]**							
Challenge	3	1.5	3.6	0.58	0.004	5.32	0.95
FAME	3.5	0.45	4	1.5	0.17	5.32	0.69
Ethanol	4	2.3	4	0.49	0.03	5.32	0.86
**6c. Log_10_ [cAMP]**							
Challenge	3	1.1	3.2	0.14	0.0009	5.59	0.98
FAME	3.5	0.36	3.0	0.92	0.86	5.59	0.38
Ethanol	3	1.4	3	1.4	0.000	7.71	1.00
**6d. AEC**							
Challenge	0.6	0.26	0.8	0.16	2.0	5.12	0.20
FAME	0.7	0.24	0.7	0.24	0.02	5.12	0.89
Ethanol	0.5	0.24	0.7	0.22	2.29	5.32	0.17

*Challenge – half of the aqueous-phase containing microcosms had been challenged with an uncharacterized microbial population derived from fuel retail UST, however substantial (≥3Log_10_ pg mL^−1^) developed in most of the unchallenged microcosms.

Previous microcosm studies performed by the current authors have been equivocal. In one experiment the aqueous-phase bioburden increased as the ethanol concentration in regular, unleaded gasoline (RUL) increased. In a second study, bioburdens developed only in microcosms with E0 or E5 (E0 – no ethanol added, E5 ethanol added to 5 % by volume) RUL but not in bottom-water under E15, E20 or E85 RUL. The current study’s AXP results suggested that although ethanol is inhibitory, the surviving population is metabolically active in fuel-associated water that contains phase-separated ethanol. Moreover, in this study, ethanol was added as a simulated diesel fuel contaminant. In previous studies, ethanol was an intentionally added RUL oxygenate.

Passman [8] reported on multiple studies in which fatty acid methyl ester (FAME) stimulated microbial growth. In the current investigation, soy-based FAME (B5 LSD and B5 ULSD) did not have a significant effect on either [cATP] or AEC. The average [cATP] was 4.0±0.72 log_10_ pg ml^−1^ in B5 diesel and 3±1.0 log_10_ pg ml^−1^ in B0 diesel. It is possible that factors other than FAME (i.e. sulphur concentration, or any of the other controlled variables) contributed to the [cATP] variability among non-replicate microcosms, thereby masking the positive impact of FAME on bioburden. Additional studies, with replicate microcosms will be needed to full assess the impact of FAME.

**Fig. 2.**
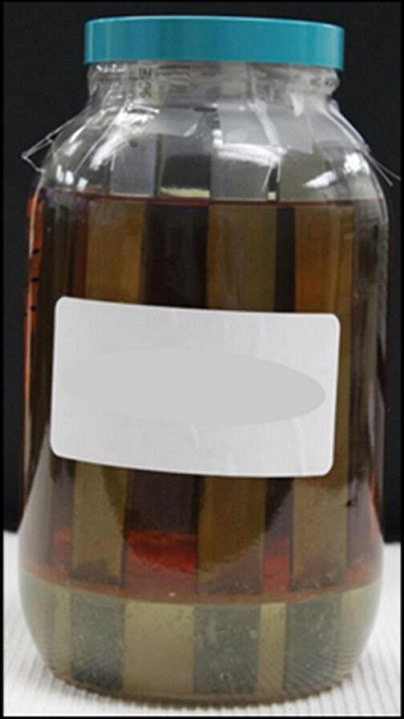
LSD over Bushnell-Hass microcosm.

A smaller subset of microcosm, aqueous-phase samples were tested at the end of weeks 12 and 18. Fig. 2 shows that during this 6 week period both the direction and magnitude of Δlog_10_ [cATP] and ΔAEC varied among microcosms. In the uninoculated, B0 ULSD microcosm – containing fuel treated with mono-acid lubricity and conductivity additives – log_10_ [cATP] decreased from 4.2 to 1.9 log_10_ pg ml^−1^ (−2.1 log_10_ pg ml^−1^). During the same period the microcosm’s AEC fell by 51 %, from 0.82 at week 12 to 0.40 at week 18. Conversely, in the inoculated microcosm containing B0 ULSD – augmented with ethanol, and mono-acid lubricity, corrosion inhibitor and conductivity additives – log_10_ [cATP] increased from 3.3 to 5.2 log_10_ pg ml^−1^ (+1.9 log_10_ pg ml^−1^). The AEC in this microcosm increased by 29 %, from 0.38 at week 12 to 0.5 at week 18. De Fuenete *et al.* [7] demonstrated that AEC can vary as populations cycle through physiological states (*homeorhesis*) and that oscillations in the range 0.2–0.3 occurred commonly. Consequently, some of the week 12 to week 18 differences could be due to these oscillations. However, homeorhesis was an unlikely explanation for the varied magnitudes or directions of the changes. Because replicate microcosms were not included in the test plan, variability among replicate microcosms could not be determined. Additional testing – using replicate microcosms – will be needed to determine how fuel system conditions affect AEC. Also, although the test microcosms were stored for 18 weeks, AECs were determined at only two time-points. In terms of the gross appearances of test microcosms and carbon-steel corrosion coupons exposed to the vapour, fuel, interface and aqueous phases, the first 2 months of exposure were the most dynamic [11]. However, there were no [cATP] or AXP tests run during this early period.

## Conclusions

For nearly 50 years, relative concentrations of ATP, ADP and AMP have been used intermittently to assess cell populations. Although this study was preliminary – using samples from an ongoing study – the results were promising. The interference removal methodology originally developed to quantify [cATP] in fuels, and other fluids in which ATP testing was historically impracticable, was used to quantify AXP – [cATP], [cADP] and [cAMP] – to determine AEC. Although the number of samples tested was limited, the results indicated that the AXP protocol was accurate and precise. The sample set on which AXP testing and AEC determinations were made was just large enough to provide tantalizing data. The results suggested that AEC could be a powerful tool for evaluating the physiological state of microbial contaminant populations and its biodeteriogenic potential in fuel-associated waters. However, more testing is needed to full validate the utility of AEC values for assessing either the impact of fuel and fuel-associated water chemistry, or remedial treatments.

## Supplementary Data

Supplementary material 1
