# Supplementary material for: The relationship between microbial community vitality and ATP bioburden in bottom waters under fuel microcosms
Source: Access Microbiol. 2023 Apr 26;5(4):acmi000411. doi: 10.1099/acmi.0.000411 (PMC10202405; doi:10.1099/acmi.0.000411)
Supplement: Supplementary material 1 [file acmi-5-411-s001.pdf]

The Relationship Between Microbial Community Vitality and ATP Bioburden in Bottoms Waters Under Fuel Microcosms

Supplementary Material

Table S-1. Diesel fuel microcosm [cATP] test result repeatability precision.

SAMPLE	1	2	3	AVG	s	C.V.
A	1.43	1.45	1.44	1.44	0.01	0.8%
B	2.45	2.45	2.44	2.45	0.00	0.2%
C	3.23	3.24	3.25	3.24	0.01	0.3%
D	4.23	4.26	4.25	4.25	0.01	0.3%
E	1.65	1.69	1.71	1.68	0.03	1.7%
F	2.82	2.80	2.85	2.82	0.02	0.8%
G	3.98	4.02	4.00	4.00	0.02	0.5%
H	3.05	3.07	3.10	3.07	0.02	0.7%
			Grand Mean	2.87		0.68%
			s	1.00		
n	24		s_r	0.17		
n-1	23		r	0.47		

Table S-2. Diesel fuel microcosm [cADP] test result repeatability precision.

SAMPLE	1	2	3	AVG	s	C.V.
A	1.40	1.48	1.55	1.48	0.08	5.1%
B	2.31	2.33	2.35	2.33	0.02	0.9%
C	2.75	2.77	2.76	2.76	0.01	0.3%
D	3.58	3.09	3.38	3.35	0.25	7.4%
E	1.75	1.73	1.77	1.75	0.02	1.1%
F	2.33	2.37	2.29	2.33	0.04	1.5%
G	3.35	3.32	3.54	3.40	0.12	3.5%
H	3.16	3.20	3.18	3.18	0.02	0.6%
			Grand Mean	2.57		2.6%
			s	0.73		
n	24		s_r	0.34		
n-1	23		r	0.95		

Table S-3. Diesel fuel microcosm [cAMP] test result repeatability precision.

SAMPLE	1	2	3	AVG	s	C.V.
A	1.63	1.66	1.77	1.69	0.08	4.5%
B	2.31	2.35	2.28	2.31	0.03	1.4%
C	2.50	2.59	2.61	2.57	0.06	2.2%
D	3.51	3.40	3.57	3.49	0.09	2.5%
E	1.75	1.77	1.76	1.76	0.01	0.5%
F	2.53	2.64	2.62	2.60	0.06	2.3%
G	2.14	2.51	2.88	2.51	0.37	14.7%
H	3.58	3.62	3.61	3.60	0.02	0.6%
			Grand Mean	2.57		3.6%
			s	0.70		
n	24		s_r	0.38		
n-1	23		r	1.08		

Table S-4. Diesel fuel microcosm AEC test result repeatability precision.

SAMPLE	1	2	3	AVG	s	C.V.
A	0.42	0.42	0.37	0.40	0.03	6.9%
B	0.57	0.54	0.55	0.55	0.01	2.1%
C	0.77	0.75	0.75	0.76	0.01	1.5%
D	0.79	0.86	0.79	0.81	0.04	4.8%
E	0.46	0.47	0.48	0.47	0.01	1.7%
F	0.64	0.58	0.61	0.61	0.03	4.7%
G	0.90	0.92	0.82	0.88	0.05	5.5%
H	0.29	0.28	0.30	0.29	0.01	2.1%
			Grand Mean	0.60		3.7%
			s	0.02		
n	24		s_r	0.19		
n-1	23		r	0.54		
